# Myogenic Determination and Differentiation of Chicken Bone Marrow-Derived Mesenchymal Stem Cells under Different Inductive Agents

**DOI:** 10.3390/ani12121531

**Published:** 2022-06-13

**Authors:** Zhen Zhou, Changbin Zhao, Bolin Cai, Manting Ma, Shaofen Kong, Jing Zhang, Xiquan Zhang, Qinghua Nie

**Affiliations:** 1 State Key Laboratory for Conservation and Utilization of Subtropical Agro-Bioresources, Lingnan Guangdong Laboratory of Agriculture, College of Animal Science, South China Agricultural University, Guangzhou 510642, China; zhenzhou@stu.scau.edu.cn (Z.Z.); zcbscau@163.com (C.Z.); bolincai@scau.edu.cn (B.C.); mamanting@stu.scau.edu.cn (M.M.); shaofenkong@163.com (S.K.); jane_zhang233@hotmail.com (J.Z.); xqzhang@scau.edu.cn (X.Z.); 2 Guangdong Provincial Key Lab of Agro-Animal Genomics and Molecular Breeding, and Key Laboratory of Chicken Genetics, Breeding and Reproduction, Ministry of Agriculture, Guangzhou 510642, China

**Keywords:** broiler, bone-derived mesenchymal stem cells, 5-azacytidine, dexamethasone, hydrocortisone, myogenic determination and differentiation

## Abstract

**Simple Summary:**

Muscle development is an important performance factor of broilers. This is the first investigation to evaluate the myogenic differentiation effect of chicken bone marrow-derived mesenchymal stem cells (BM-MSCs) induced by 5-azacytidine (5-Aza). qRT-PCR was performed to compare myogenic determination and differentiation of chicken BM-MSCs under different inductive agents. Transcriptome sequencing and a Western blot were performed to further confirm the myogenic effect induced by 5-Aza. In conclusion, our study indicated that BM-MSCs demonstrate better myogenic differentiation potential under 5-day treatment with 5-Aza. Our findings lay the foundation for constructing a myogenic determination and differentiation model of chicken BM-MSCs.

**Abstract:**

Poultry plays an important role in the meat consumer market and is significant to further understanding the potential mechanism of muscle development in the broiler. Bone marrow-derived mesenchymal stem cells (BM-MSCs) can provide critical insight into muscle development due to their multi-lineage differentiation potential. To our knowledge, chicken BM-MSCs demonstrate limited myogenic differentiation potential under the treatment with dexamethasone (DXMS) and hydrocortisone (HC). 5-azacytidine (5-Aza), a DNA demethylating agent, which has been widely used in the myogenic differentiation of BM-MSCs in other species. There is no previous report that applies 5-Aza to myogenic-induced differentiation of chicken BM-MSCs. In this study, we evaluated the myogenic determination and differentiation effect of BM-MSCs under different inductive agents. BM-MSCs showed better differentiation potential under the 5-Aza-treatment. Transcriptome sequence analysis identified 2402 differentially expressed DEGs including 28 muscle-related genes after 5-Aza-treatment. The DEGs were significantly enriched in Gene Ontology database terms, including in the cell plasma membrane, molecular binding, and cell cycle and differentiation. KEGG pathway analysis revealed that DEGs were enriched in myogenic differentiation-associated pathways containing the PI3K-Akt signaling pathway, the TGF-β signaling pathway, Arrhythmogenic right ventricular cardiomyopathy, dilated cardiomyopathy, and hypertrophic cardiomyopathy, which suggested that BM-MSCs differentiated into a muscle-like phenotype under 5-Aza-treatment. Although BM-MSCs have not formed myotubes in our study, it is worthy of further study. In summary, our study lays the foundation for constructing a myogenic determination and differentiation model in chicken BM-MSCs.

## 1. Introduction

The poultry industry plays an important role in the consumer market due to its high-quality meat and eggs [1,2]. In recent years, poultry meat has gradually become predominant in the meat market due to the African swine fever, and the world production of poultry meat is expected to increase by 20.3 million tons between 2017 and 2029 in the future [3]. With the increase in poultry meat demands, the quality of poultry meat gains importance. Thus, exploring a potential muscle development mechanism is significant for the further development of the poultry industry.

Bone morrow-derived mesenchymal stem cells (BM-MSCs), one kind of cell possessing multi-lineage differentiation potential, are widely used as a good model to further understand the potential molecule mechanism of muscle development, fat deposition, and skeletal growth [4]. BM-MSCs are easier to isolate and do not demand strict culture conditions in comparison to embryonic stem cells and induced pluripotent stem cells, making BM-MSCs an attractive research object. Recent applications of BM-MSCs are focused on injury therapy, immunomodulatory, spontaneous differentiation of connective tissue, and induced differentiation of other cell types including myoblasts, osteocytes, adipocytes, and nerve cells in vitro [5,6,7,8].

Different agents can induce BM-MSCs to differentiate into different cell types. For example, the adipogenic potential of chicken BM-MSCs can be activated by a combination of agents containing 3-isobutyl-1-methylxanthine, dexamethasone (DXMS), insulin, and indomethacin. Osteogenic potential can be activated by β-glycerophosphate, ascorbate, insulin, and dexamethasone. However, to our knowledge, no attention has been paid to inducing myogenic differentiation of BM-MSCs in chicken. Adhikari et al., demonstrated that BM-MSCs showed a myogenic differentiation potential under treatment with DXMS and hydrocortisone (HC) [9]. Beyond that, no further relevant studies have been reported.

5-azacytidine (5-Aza), one kind of DNA methylation inhibitor, is an epigenetic drug that can upregulate gene expression by reversing the repressive state of DNA hypermethylation [10]. In the field of stem cells, 5-Aza is wildly applied to induce MSCs to differentiate into myoblasts, cardiomyocytes, and myotubes for cardiomyopathy injury therapy [11,12,13,14]. Thus, 5-Aza has enormous potential to construct a myogenic differentiation model. However, there is no relevant report about applying 5-Aza to chicken BM-MSCs.

**Figure 1:**
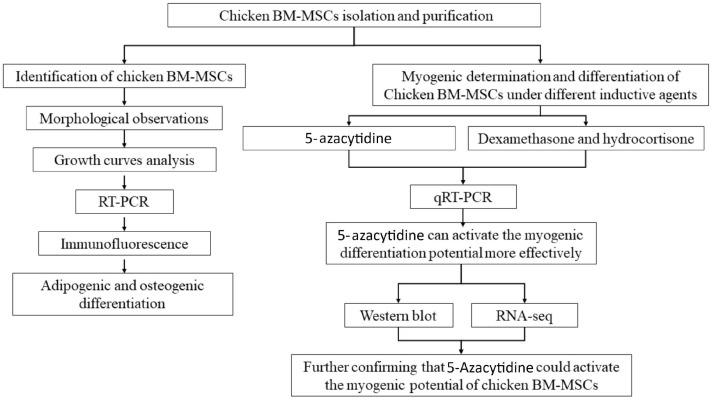
The experimental flow of this study. Experiments in this study can be divided into three parts, including the isolation of chicken BM-MSCs, identification of chicken BM-MSCs, and myogenic differentiation of chicken BM-MSCs under different inductive agents.

In this study, we applied 5-Aza to induce myogenic differentiation in comparison with DXMS and HC. We indicated that BM-MSCs showed better differentiation potential under the 5-Aza-treatment. The experimental flow is shown below (Figure 1).

## 2. Materials and Methods

### 2.1. Ethics Statement

The animal experiment performed in this study satisfied the requirements of the Institutional Animal Care and Use Committee at the South China Agricultural University (approval ID: 2021-C018).

### 2.2. Animal and Cells

The 1-day-old chickens were purchased from Xufeng Farming Co., Ltd. (Kaiping, China). The chicken macrophage cell line (HD11) was from the Guangdong Provincial Key Laboratory of Animal Health Aquaculture and Environmental Control.

### 2.3. Primary BM-MSCs Isolation

**Figure 2:**
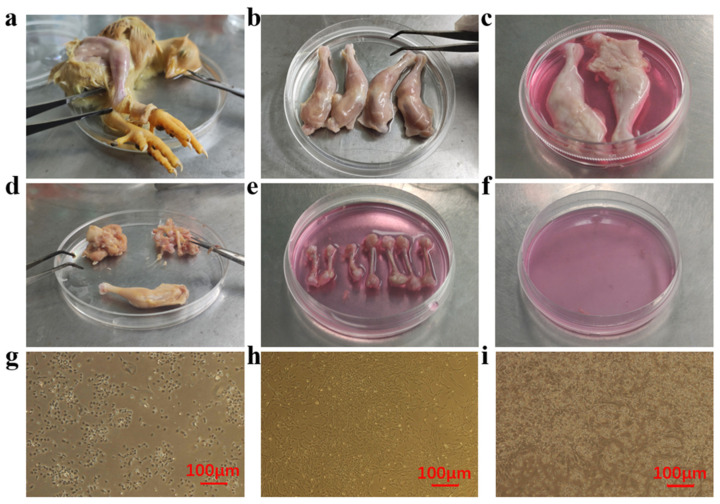
Isolation of chicken BM-MSCs. (**a**) The chicken was soaked in 75% alcohol to dissect legs and then (**b**) soaked the legs in 75% alcohol and (**c**) serum-free DMEM sequentially. (**d**) Removing muscles and connective tissues stuck to femurs and tibia. (**e**) Femurs and tibia were soaked in serum-free DMEM and cut off both sides of the epiphysis to expose the bone marrow cavity. (**f**) The bone marrow cavity was flushed by serum-free DMEM to collect cells. Finally, a differential adhesion method was performed to purify BM-MSCs. The length of the first differential adhesion time affected the purity of BM-MSCs. Morphological observations showed that (**g**) 2 h differential adhesion did not collect enough BM-MSCs; (**h**) 4 h differential adhesion collected enough BM-MSCs; (**i**) 24 h differential adhesion resulted in low cell purity of BM-MSCs.

Primary BM-MSCs were isolated from the femur and tibia of 1-day-old chickens. Chickens were killed and sterilized with 75% alcohol for 5–10 min (Figure 2a). Chicken legs were dissected on a clean bench, soaked in 75% alcohol, and then kept in serum-free Dulbecco’s Modified Eagle Medium (DMEM) (ThermoFisher, Waltham, MA, USA) (Figure 2b,c). The muscles and connective tissues attached to the femur and tibia bones were removed, followed by the removal of bone epiphysis to expose the bone marrow cavity (Figure 2d,e). The marrow cavity was flushed using serum-free DMEM to collect whole cells (Figure 2f). The liquid was filtered with a 70 μm sterile strainer (Corning, New York, NY, USA) and the filtrate was centrifuged at 1000 rpm for 5 min to get rid of plasma and lipids. The supernatant was discarded and a growth medium (GM, DMEM/F12 (ThermoFisher, Waltham, MA, USA) containing 10% fetal bovine serum (FBS) (ThermoFisher, Waltham, MA, USA)) was used to resuspend cells. The medium was completely changed after 4 h and 24 h. The adherent BM-MSCs were marked as passage 0 (P0) or primary cells (Figure 2h). When the time of the first differential adhesion was reduced to 2 h, there were not enough BM-MSCs collected in order to culture (Figure 2g). When the time was increased to 24 h, too many other cells were adherent and the purity of BM-MSCs decreased (Figure 2i).

### 2.4. BM-MSCs Culture and Subculture

Cultures were incubated at 37 °C in a humidity incubator containing 5% CO_2_. BM-MSCs required subculture when reaching 90% confluence. Cells were washed by PBS (ThermoFisher, Waltham, MA, USA), and dissociated with 0.25% Trypsin-EDTA (ThermoFisher, Waltham, MA, USA) for 2.5 min. GM was then added to stop dissociation and the cell suspension was collected to be centrifuged at 1000 rpm for 5 min. Finally, the cell layer was resuspended and then subcultured in new cell culture dishes. The subcultured BM-MSCs were named passage 1 (P1), and subsequent passaged cells were named P2, P3, or P4. Cell viability was checked during the passages by the Countstar Automated Cell Counter (ALIT Life Science, Shanghai, China).

### 2.5. Growth Curve Assay

BM-MSCs were cultured in 24-well plates with 2 × 10^4^ cells/mL per well. In the next 8 days, cells were dissociated by 200 μL 0.25% trypsin-EDTA for 2.5 min. 400 μL GM was added to stop dissociation and prepare cell suspension. The Countstar Automated Cell Counter was used to calculate the cell number of 3-well BM-MSCs daily. Every well was counted three times to obtain the mean value.

### 2.6. RNA Extraction and cDNA Synthesis

Total RNA was extracted with RNAiso Plus (Takara, Kyoto, Japan) and the HiPure Universal RNA Mini Kit (Magen, Guangzhou, China) following the manufacturer’s protocol. cDNA was synthesized using MonScript™ 5× RTIII All-in-One Mix kits (Monad, Shanghai, China) for reverse transcription.

### 2.7. Reverse Transcription PCR (RT-PCR)

BM-MSCs were harvested to extract the total RNA and synthesize cDNA. cDNA samples were subjected to PCR amplification using 2 × EasyTaq PCR SuperMix (TransGen Biotech, Beijing, China). The thermocycling parameter was at 98 °C for 3 min, followed by 35 cycles of 98 °C for 10 s, 56 °C for 10 s, and 72 °C for 10 s, and followed by 72 °C for 2 min. HD11 was employed as the positive control. PCR products were separated by 1.5% agarose gel electrophoresis to visualize the band, and it was detected using GoldView II Nuclear Staining Dyes (Solarbio, Beijing, China). Primers for each marker gene were designed and checked for target identity using the NCBI database. The information on primers used for RT-PCR assays was presented in Table S1.

### 2.8. Immunofluorescence (IF)

BM-MSCs were plated in a 12-well plate for IF assay to detect cell surface markers. HD11 was employed as a positive control in the detection of CD45.

Cells were washed with PBS to remove GM. Fixation and permeabilization were performed at room temperature. Cells were fixed in 4% formaldehyde for 20 min. The fixed cells were incubated with 0.1% Triton X-100 (diluted with PBS) for 15 min. After that, cells were blocked with 10% goat serum (Beyotime, Shanghai, China) for 30 min and incubated overnight with primary antibody (diluted with Immunol staining primary antibody dilution buffer (Beyotime, Shanghai, China)). After rinsing with PBS three times, the cells were incubated with the secondary antibody (1:1000; Abcam, Cambridge, UK) for 1 h at room temperature. Finally, cells were incubated with a 10% DAPI staining solution (Beyotime, Shanghai, China) for 5 min.

The information of antibodies used in IF was listed as follows: rabbit anti CD29 polyclonal antibody (LS-C413122, LSBio, Seattle, WA, USA, 1:400), rabbit anti CD105 polyclonal antibody (bs-0579R, Bioss, Zhuhai, China, 1:400), rabbit anti CD166 polyclonal antibody (bs-1251R, Bioss, Zhuhai, China, 1:400), mouse anti CD45 monoclonal antibody (MA5-28682, Invitrogen, Waltham, MA, USA, Monoclonal, 1:400), Goat anti-Mouse lgG H&L polyclonal (FITC) (ab6785, Abcam, Cambridge, UK, 1:40,000), and Goat anti-Rabbit lgG H&L polyclonal (FITC) (ab6717, Abcam, Cambridge, UK, 1:40,000).

### 2.9. Induction of Adipogenic Differentiation

Adipogenic medium (AM) containing DMEM/F12 (10% FBS), 0.5 mM 3-isobutyl-1-methylxanthine (Sigma-Aldrich, Milwaukee, MI, USA), 1 μM dexamethasone (Sigma-Aldrich, Milwaukee, MI, USA), 10 μg/mL insulin (Sigma-Aldrich, Milwaukee, MI, USA), and 200 μM indomethacin (Sigma-Aldrich, Milwaukee, MI, USA) were used to induce the adipogenic differentiation of BM-MSCs for 3 days, 6 days, 9 days, or 12 days. The AM was changed every 3 days for 12 days.

### 2.10. Induction of Osteogenic Differentiation

Osteogenic medium (OM) containing DMEM/F12 (10% FBS), 10 mM β-glycerophosphate (Sigma-Aldrich, Milwaukee, MI, USA), 50 μg/mL ascorbate (Sigma-Aldrich, Milwaukee, MI, USA), 10 μg/mL insulin (Sigma-Aldrich, Milwaukee, MI, USA), and 1 μM dexamethasone (Sigma-Aldrich, Milwaukee, MI, USA) were used to induce the osteogenic differentiation of BM-MSCs for 21 days. The OM was changed every 3 days for 21 days.

### 2.11. Induction of Myogenic Differentiation

Two types of myogenic mediums were used in this study. One is the 5-Aza-myogenic medium containing DMEM/F12 (2% horse serum) and 10 µM 5-Aza (Macklin, Shanghai, China). Another is DXMS-HC-myogenic medium containing DMEM/F12 (2% horse serum) and 0.1 µM DXMS (Sigma-Aldrich, Milwaukee, MI, USA), 50 µM HC (Sigma-Aldrich, Milwaukee, MI, USA).

5-Aza-myogenic medium and DXMS-HC-myogenic medium were used to induce the myogenic differentiation of BM-MSCs for 3 days, then the medium was changed with DMEM/DMEM/F12 (2% horse serum) for continuous 2-day or 4-day induction.

### 2.12. Oil Red O Staining

After being treated with AM for 12 days, the cultures were washed with PBS and fixed in 4% formaldehyde for 20 min. The cultures were then dyed with oil red O solution (BBI, Shanghai, China) for 60 min at room temperature and then washed three times with PBS, according to the manufacturer’s specification. After washing, a fluorescence inverted light microscope (Leica DMi8, Wetzlar, Germany) was used to capture images.

### 2.13. Alizarin Red Staining and Alkaline Phosphatase Assay

After being treated with OM for 21 days, the cultures were stained with Alizarin Red (Solarbio, Beijing, China) for detection of mineralization. The alkaline phosphatase was detected using the Alkaline Phosphatase Assay Kit (Beyotime, Shanghai, China) on 21 days of OM treatment.

### 2.14. Western Blot

RIPA buffer (Beyotime, Shanghai, China) containing Phenylmethanesulfonyl fluoride (Beyotime, Shanghai, China) was used to lyse cells. The homogenate was centrifuged at 13,000× *g* for 10 min to collect the supernatant. Protein concentration was estimated by BCA assay with the BCA Protein Assay Kit (Beyotime, Shanghai, China). Proteins were separated in 12% SDS-PAGE, transferred onto a PVDF membrane, and then probed with antibodies following standard procedures. After washing with TBST three times, the membranes were incubated with 5% skimmed milk powder in TBST at room temperature for 2 h and then primary antibodies at 4 °C overnight. After washing with TBST three times, the membranes were incubated with secondary antibodies at room temperature for 1 h. After washing with TBST three times, the Bands were visualized using ECL reagents (Beyotime, Shanghai, China) and analyzed with a gel analysis system. The gray values were calculated by Image J software.

The following antibodies and their dilutions were used in the Western blot: rabbit anti-PPAR gamma polyclonal antibody (bs0530R, Bioss, Zhuhai, China, 1:1000); rabbit anti-CEBP alpha polyclonal antibody (bs-24540R, Bioss, Zhuhai, China, 1:1000); rabbit anti-beta-actin polyclonal antibody (bs-0061R, Bioss, Zhuhai, China, 1:5000); MyoD1 monoclonal antibody (PM2147a, GenePharma, Suzhou, China, 1:100); MyoG polyclonal antibody (orb6492, Biorbyt, Cambridge, UK, 1:500); HRP, Goat Anti-Rabbit IgG polyclonal (A21020, Abbkine, Wuhan, China, 1:10,000); and HRP, Goat Anti-Mouse IgG polyclonal (A21010, Abbkine, Wuhan, China, 1:10,000).

### 2.15. Quantitative Real-Time PCR (qRT-PCR)

Total RNA extraction and cDNA synthesis followed the method described above. cDNA samples were subjected to ChamQ Universal SYBR qPCR Master Mix (Vazyme Biotech, Nanjing, China) following the manufacturer’s protocol. The 2^−ΔΔCt^ method and internal normalization were used to analyze quantification results. *GAPDH* was employed as the housekeeper gene. Primer information is presented in Table S1.

### 2.16. Transcriptome Sequencing

Treated and control cells were collected to extract total RNA using the Trizol reagent kit (Invitrogen, Carlsbad, CA, USA) according to the manufacturer’s protocol. Three biological replicates were performed for both groups. RNA quality was assessed on an Agilent 2100 Bioanalyzer (Agilent Technologies, Palo Alto, CA, USA) and checked using RNase-free agarose gel electrophoresis. Eukaryotic mRNA was then enriched by Oligo(dT) beads. The enriched mRNA was fragmented into short fragments using fragmentation buffer and reverse transcribed into cDNA with random primers. Second-strand cDNA was synthesized by DNA polymerase I, RNase H, dNTP, and buffer. Then the cDNA fragments were purified with a QiaQuick PCR extraction kit (Qiagen, Venlo, The Netherlands), end-repaired, poly(A) added, and ligated to Illumina sequencing adapters. The ligation products were size-selected by agarose gel electrophoresis, PCR amplified, and sequenced using Illumina HiSeq2500 by the Gene Denovo Biotechnology Co. (Guangzhou, China).

RNAs differential expression analysis was performed by DESeq2 software between two groups. The genes with the parameter of false discovery rate below 0.05 and absolute fold change ≥2 were considered as differentially expressed genes (DEGs). The sequencing data reported in this study were archived in the NCBI SRA database with the accession number PRJNA756416.

### 2.17. GO and KEGG Enrichment Analysis

Gene ontology (GO) enrichment analysis provided all GO terms that significantly enriched in DEGs compared to the genome background and filtered the DEGs that correspond to biological functions. Firstly, all DEGs were mapped to GO terms in the GO database (http://www.geneontology.org/, accessed on 2 August 2021). Gene numbers were calculated for every term, and significantly enriched GO terms in DEGs were defined by a hypergeometric test. The calculated *p*-value was entered through false discovery rate correction, using a false discovery rate ≤ 0.05 as a threshold. GO terms meeting this condition were defined as significantly enriched GO terms.

KEGG enrichment analysis identified significantly enriched metabolic pathways or signal transduction pathways in DEGs compared with the whole genome background. The calculated *p*-value was gone through false discovery rate correction, taking false discovery rate ≤ 0.05 as a threshold. Pathways meeting this condition were defined as significantly enriched pathways in DEGs.

### 2.18. Statistical Analysis

All experiments in this study were repeated 3 times at least to ensure repeatability and all data are expressed as means ± SEM. An independent sample *t*-test was used to compare differences between the two groups and *p* < 0.05 was considered statistically significant between the groups. All statistical analyses were performed using SPSS 23.0 for Window (SPSS, Inc., Chicago, IL, USA). Symbol “*” and “**” indicate a significant difference at *p* < 0.05 and *p* < 0.01, respectively.

## 3. Results

### 3.1. BM-MSCs Showed Normal Morphological Characteristic and Growth Curve

BM-MSCs had three cell shapes: round cells, spindle cells, and polygonal cells during proliferation. After subculture, round cells were in the majority at the beginning. Subsequently, the spindle and polygonal cells increased, and round cells almost disappeared. Sub-cultured BM-MSCs reached 50% confluence in 4 days, 80% confluence in 6 days and 100% confluence in 8 days (Figure S1a). In the growth curve, BM-MSCs showed a latent phase of 1–4 days and a logarithmic growth phase of 4–7 days. When culture was up to 8 days, the number of cells began to decrease. The reason may be due to excessive cell density and insufficient nutrients probably (Figure S1b). The results demonstrated that BM-MSCs isolated by the 4 h differential adhesion method showed normal growth characteristics.

### 3.2. Primary BM-MSCs Expressed Special Cell Surface Markers Genes and Protein

**Figure 3:**
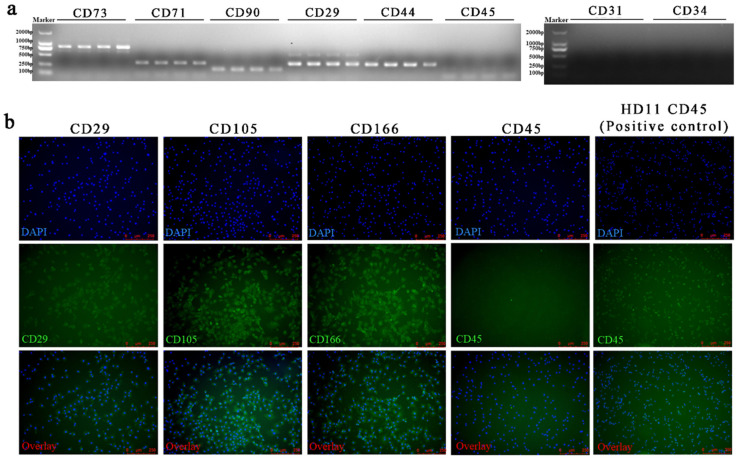
Identification of chicken BM-MSCs. Cell surface markers of BM-MSCs were detected by RT-PCR and IF. (**a**) The RT-PCR results showed that BM-MSCs were positive for *CD73*, *CD71*, *CD90*, *CD29*, and *CD44* but negative for *CD45*, *CD31*, and *CD34*. Every cell surface marker was detected four times to ensure repeatability. (**b**) The IF results showed that BM-MSCs expressed CD29, CD105, and CD166 but not expressed CD45. HD11 was employed as the positive control of CD45.

The RT-PCR analysis revealed that BM-MSCs expressed *CD73*, *CD71*, *CD90*, *CD29*, *CD44*, but not expressed *CD45*, *CD31*, *CD34* (Figure 3a). The IF analysis further demonstrated that BM-MSCs expressed CD29, CD105, and CD166 but not CD45 (Figure 3b). CD73, CD71, CD90, CD29, CD44, CD105, and CD166 were considered the positive markers of BM-MSCs in several studies. CD45 is a pan-leukocyte marker; CD34 marks primitive hematopoietic progenitors and endothelial cells; CD31 is a marker for endothelial cells. CD45, CD34, and CD31 were used as negative markers to identify the purity of BM-MSCs. These results suggested that BM-MSCs isolated in this study possessed molecular markers of BM-MSCs and homogeneity.

### 3.3. BM-MSCs Demonstrated Adipogenic and Osteogenic Differentiation 

**Figure 4:**
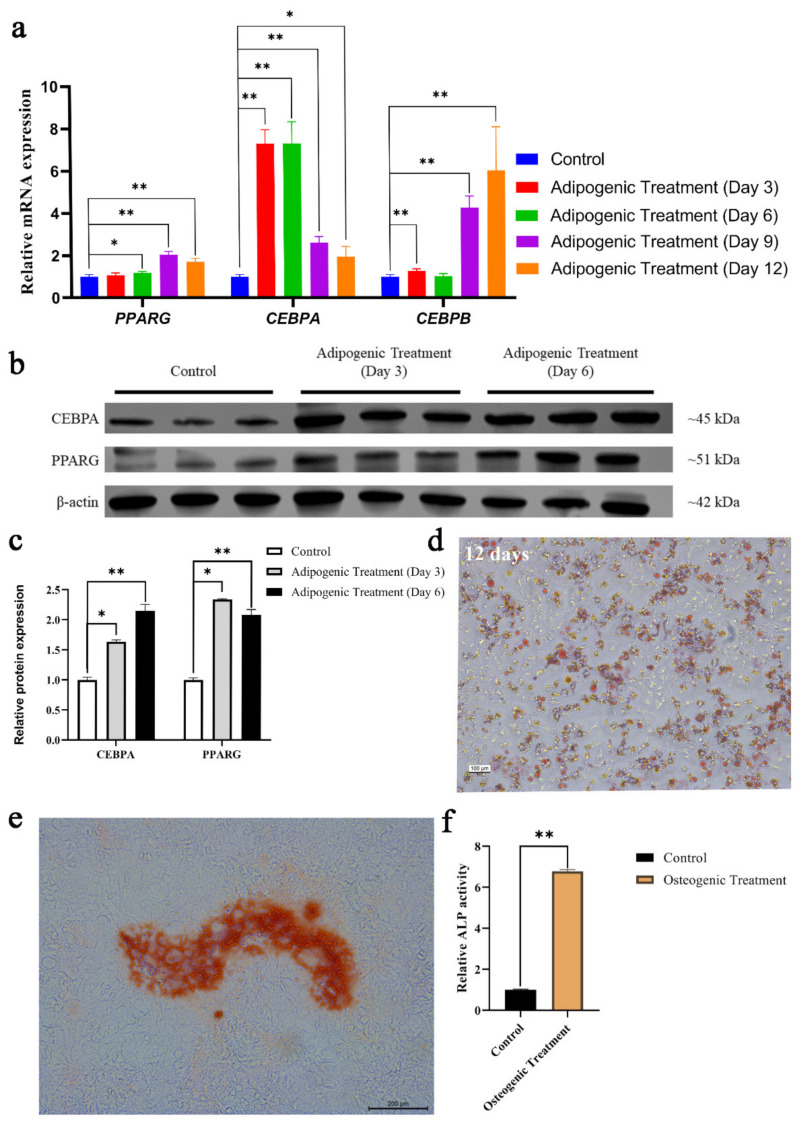
Induction of adipogenic and osteogenic differentiation of chicken BM-MSCs. (**a**) Adipogenic genes were detected using qRT-PCR after adipogenic treatment for 3 days, 6 days, 9 days, or 12 days. (**b**) Western blot was used to detect the adipogenic protein after adipogenic treatment for 3 days and 6 days. (**c**) Adipogenic protein expression assessed by Western blot analysis is expressed relative amount to β-actin. (**d**) Oil red O staining confirmed the adipogenic differentiation of BM-MSCs. (**e**) Alizarin Red Staining showed that mineralized bone nodules have formed under osteogenic treatment. (**f**) ALP activity increased significantly after osteogenic treatment compared with normal BM-MSCs. Symbol “*” and “**” indicate a significant difference at *p* < 0.05 and *p* < 0.01, respectively.

BM-MSCs treated with an adipogenic medium expressed a higher level of CEBPA, CEBPB, and PPARG mRNA in comparison to normal BM-MSCs (Figure 4a). However, PPARG expression was not significantly higher after 3-day adipogenic treatment and the expression of CEBPB was not significantly different between 6-day adipogenic treatment and control cells (Figure 4a). To further confirm the ability of adipogenic differentiation, Western blot and oil red O staining were employed to measure the differentiated effect. The results showed that the adipogenic protein expression of the 3-day-treated and 6-day-treated group was significantly higher than control cell (Figure 4b,c), and oil red O staining confirmed adipogenesis of 12-day-induced differentiation (Figure 4d). In addition to adipogenic differentiation, osteogenic differentiation is also available. Alizarin red staining and alkaline phosphatase assay were performed to measure the osteogenic effect. The results showed that mineralized bone nodules were formed (Figure 4e), and ALP activity was significantly increased in 21-day osteogenic treatment (Figure 4f).

### 3.4. Myogenic Determination and Differentiation Potential of Chicken BM-MSCs under Different Inductive Agents

**Figure 5:**
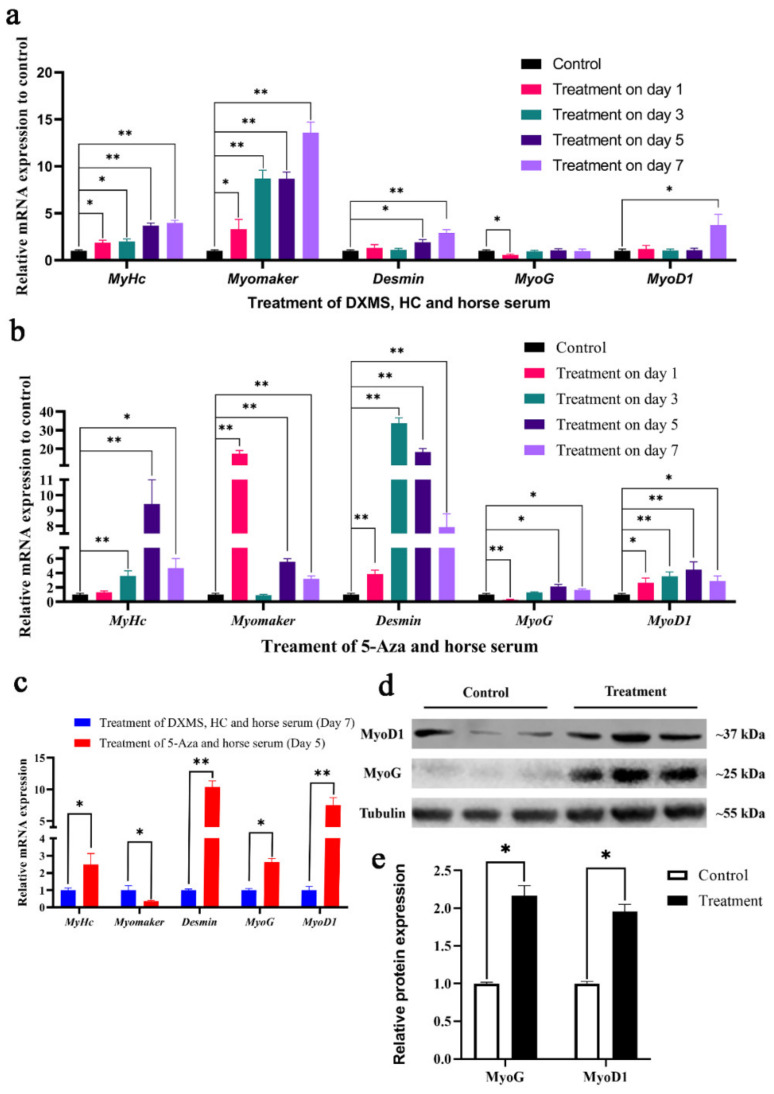
Induction of myogenic differentiation in chicken BM-MSCs under different inductive agents. Muscle-related genes were detected using qRT-PCR after (**a**) DXMS and HCor and (**b**) 5-Aza treatment for 1 day, 3 days, 5 days, or 7 days. (**c**) Based on the qRT-PCR data, the 5-Aza-treated group showed a better myogenic effect in comparison with the DXMS-HC-treated group. (**d**) Western blot results showed that MyoD1 and MyoG were significantly upregulated with the treatment of 5-Aza for 5 days. (**e**) Myogenic protein expression assessed by Western blot analysis is expressed relative amount to Tubulin. Symbol “*” and “**” indicate a significant difference at *p* < 0.05 and *p* < 0.01, respectively.

BM-MSCs were treated with 5-Aza or DXMS, HC for 1, 3, 5, and 7 days, respectively. Both myogenic treatments induced dramatic morphologic changes (Figure S2). qRT-PCR was employed to assay the mRNA expression of muscle-related genes *MyHC*, *Myomaker*, *MyoD1*, *MyoG*, and *Desmin*. According to qRT-PCR results, chicken BM-MSCs did demonstrate myogenic differentiation potential, which is consistent with what Adhikari et.al reported (Figure 5a). *MyHc* and *Myomaker* were significantly high expression during 7-day induction and reached the highest expression level on day 7. *Desmin* was significantly upregulated until day 5 and also reach the highest expression level on day 7. *MyoD1* was not significantly upregulated until day 7. Surprisingly, the expression of *MyoG* was even downregulated after 1-day treatment and showed no significant difference in the latter induced process. Based on these results, we considered that 7 days was the best induction time in DXMS-HC treatment, but DXMS-HC treatment only activated a limited myogenic differentiation potential in BM-MSCs.

By contrast, BM-MSCs demonstrated better myogenic differentiation potential under 5-Aza treatment (Figure 5b). In the 1-day treatment stage, the mRNA of *Myomaker* reached the highest expression among all induced stages. *Desmin* and *MyoD1* also significantly high expression compared to control. But the expression of *MyHc* showed no significant difference. *MyoG* was also significantly downregulated, which was consistent with DXMS-HC treatment. In the 3-day treatment, *Myomaker* and *MyoG* showed no significant difference, but *Desmin* reached the highest expression. *MyHc* and *MyoD1* expression remained high. Interestingly, all five muscle-related genes were significantly upregulated under the 5-day and 7-day treatments. Between these two stages, the 5-day treatment group showed higher relative expression. Based on the above results, we believed that 5 days was the best induced time for 5-Aza treatment. In addition, it demonstrated better myogenic differentiation potential compared with the 7-day-DXMS-HC treatment in our study (Figure 5c).

To further confirm the myogenic effect of the 5-day-5-Aza treatment, a Western blot was performed to detect the protein expression of MyoD1 and MyoG. The result showed that MyoD1 and MyoG expression in the 5-Aza treatment was about two times more than that of control (Figure 5d,e). It indicated that BM-MSCs did show a certain potential of myogenic differentiation after 5-Aza treatment for 5 days.

### 3.5. Transcriptome Sequencing Revealed Great Change in Gene Expression of BM-MSCs under 5-Aza Treatment

**Figure 6:**
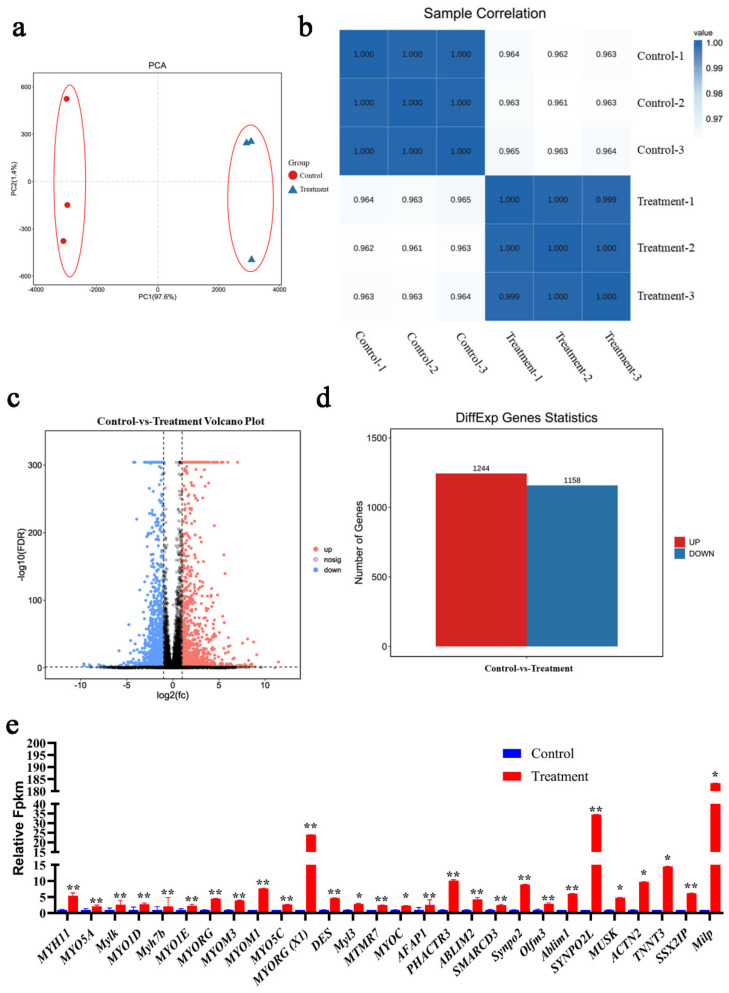
Transcriptome sequencing analysis of BM-MSCs treated with 5-Aza. (**a**) PCA of transcriptomic variation. Shapes denoted different groups of the cell samples; colored dots indicated different samples. (**b**) Correlation analysis of different samples. (**c**) The volcano plots map of all DEGs between control cells and treated cells. (**d**) The number of up-regulated and down-regulated DEGs and (**e**) 28 muscle-related DEGs were up-regulated by the treatment of 5-Aza and horse serum in comparison to control cells. Symbol “*” and “**” indicate a significant difference at *p* < 0.05 and *p* < 0.01, respectively.

Transcriptome sequencing was performed to analyze the DEGs between normal BM-MSCs and 5-Aza-treated BM-MSCs (day 5). Sequencing and mapping information were listed in Table S2. The principal component analysis (PCA) result demonstrated that the treated group and control group were distinctly clustered (Figure 5a), which was also supported by the sample correlation results (Figure 6b), suggesting acceptable repeatability in each and significant difference under 5-Aza treatment.

Based on the sequencing data, a total of 2402 DEGs were found, of which 1244 were up-regulated and 1158 were down-regulated (Figure 6c,d). Among these DEGs, we found that 28 muscle-related genes were upregulated after treatment (Figure 6e) (the detailed information of 28 genes was listed in Table S3. It suggested that 5-Aza can activate the myogenic differentiation potential of chicken BM-MSCs.

### 3.6. GO and KEGG Enrichment of DEGs between Myogenic-Treated BM-MSCs and Normal BM-MSCs

**Figure 7:**
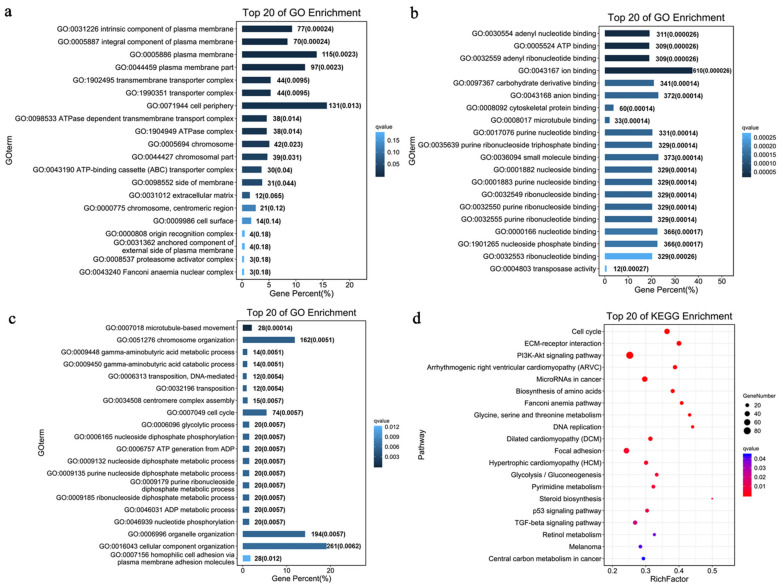
GO and KEGG enrichment of DEGs in transcriptome sequencing analysis. GO enrichment analysis of DEGs in (**a**) cellular component, (**b**) molecular function, and (**c**) biological process. (**d**) KEGG pathway enrichment analysis of enriched DEGs.

GO and KEGG enrichment analyses were performed to further explore the function of 2402 identified DEGs. In GO enrichment analysis, 2402 DEGs were enriched in a total of 519 GO terms, including cellular component (CC), biological process (BP), and molecular function (MF). DEGs were significantly enriched in CC which mainly includes plasma membrane-related terms (Figure 7a), MF which includes binding-related terms (Figure 7b), and BP which mainly includes cell cycle and differentiation-related terms (Figure 7c).

The KEGG pathway analysis results showed that DEGs were enriched in 58 pathways at *p* < 0.05. Among the top 20 KEGG enrichment pathways, cell differentiation-associated signaling pathways were enriched, such as the PI3K-Akt signaling pathway and the TGF-β signaling pathway (Figure 6i). It means that 5-Aza possibly induces myogenic differentiation of cMSCs via the PI3K-Akt signaling pathway and TGF-β signaling pathway. Furthermore, arrhythmogenic right ventricular cardiomyopathy, dilated cardiomyopathy, and hypertrophic cardiomyopathy was also enriched, which were associated with muscle development. The above results suggested that 5-Aza did have the potential to induce chicken BM-MSCs to differentiate into muscle lineage.

## 4. Discussion

With the rapid development of the poultry industry, high-quality poultry meat products are desired by consumers. Researchers need to improve poultry meat quality by exploring the mechanism of muscle development [15,16]. BM-MSCs, which have the potential for myogenic differentiation, can provide critical insights to further understand the potential molecular mechanism in muscle development [9]. This study is the first investigation to evaluate the myogenic effect of chicken BM-MSCs under different inductive agents. We demonstrated that 5-Aza treatment could significantly promote the myogenic differentiation potential of chicken BM-MSCs.

The differential adhesion method is one of the most common methods to isolate and purify BM-MSCs. To improve the purity of BM-MSCs, a modified method for rapid purification was used in this study. The culture medium was completely exchanged at 4 h and 24 h. CD73, CD90, and CD105 are known as 5′-Nucleotidase, Thy-1 cell surface antigen, and endoglin, respectively, which were considered positive markers of BM-MSCs by the International Society for Cellular Therapy (ISCT) [17]. CD45 was a pan-leukocyte marker and CD34 was used to mark primitive hematopoietic progenitors and endothelial cells [17,18]. In addition, transferrin receptor (CD71), integrin subunit beta 1 (CD29), CD44 and (negative) were widely used to identify chicken BM-MSCs [9,19,20]. Recently, CD166 activated leukocyte cell adhesion molecule, was reported to use as an expression marker for BM-MSCs characterization [21]. Based on this research, CD73, CD71, CD90, CD29, CD44, CD105, and CD106 were used as positive markers, and CD45, CD31, and CD34 were selected to use as negative markers to identify chicken BM-MSCs in this study. According to the results of qRT-PCR and IF detection, we detected the presence of BM-MSCs surface markers CD73, CD71, CD90, CD29, CD44, CD105, CD106, and a lack of CD45, CD31, and CD34 expression. These results suggested that BM-MSCs isolated in this study matched the minimal criteria for defining MSCs and other relevant studies about BM-MSCs identification.

BM-MSCs’ myogenic differentiation is an important appliance to provide critical insight into muscle development. In 2018, Adhikari et al. induced the myogenic differentiation of BM-MSCs for the first time using DXMS and HC [9]. They confirmed that BM-MSCs demonstrated the potential of myogenic phenotypes, which provide a new potential method to induce the myogenic differentiation of BM-MSCs. Based on their research, we also evaluated the myogenic differentiation potential of BM-MSC under DXMS-HC treatment and found that BM-MSCs certainly showed myogenic potential. This is consistent with their study.

Except for DXMS and HC, DNA demethylating agent 5-Aza has shown the ability to activate the myogenic phenotypes of BM-MSCs in 1995 [14]. Based on previous studies, 5 and 10 µM were chosen as the 5-Aza induced concentration from the beginning. After performing the induced experiment, we revealed that 10 µM-5-Aza treatment could activate a better myogenic differentiation effect (data not shown). In this study, we evaluated the myogenic differentiation potential of BM-MSCs under 10 µM-5-Aza treatment. qRT-PCR results showed that the mRNA expression level of muscle-specific genes was up-regulated when BM-MSCs were treated with 5-Aza for 5 and 7 days, which were consistent with data in the previous studies [22,23,24]. Our data confirmed that BM-MSCs would show the potential of myogenic differentiation under 5-Aza treatment. Moreover, we demonstrated that BM-MSCs reached the best myogenic effect under 5-Aza treatment for 5 days. This finding is not completely consistent with previous studies which have suggested that the best myogenic effect was reached in the treatment of 5-Aza for 14, 21, and 40 days [13,22,24]. Zhang et al. evaluated the best time of myogenic differentiation of human MSCs, and they demonstrated that treating with 5-Aza for 7 days was more effective [24]. This inconsistency may be due to the different sources for BM-MSCs. In chicken, we believe that MSCs treated with 5-Aza for 5 days were more effective. The 5-Aza-treated BM-MSCs showed higher myogenic differentiation potential than DXMS-HC treatment in this study. However, it cannot be denied that these two methods are both potential methods to establish the myogenic differentiation model of BM-MSCs.

Transcriptome analysis was performed to further analyze the DEGs between 5-Aza-treated BM-MSCs and control BM-MSCs. The repeatability and differences were examined by PCA, and correlation analysis, and the results showed that there was good repeatability among each replicate sample and distinction between these two groups. The transcriptome sequence analysis showed that 1244 up-regulated DEGs and 1158 down-regulated DEGs were identified. These 1244 up-regulated genes included 28 muscle-related genes, containing *MYH11*, *MYO5A*, *Mylk*, *MYO1D*, *Myh7b*, *MYO1E*, *MYORG*, *MYOM3*, *MYOM1*, *MYO5C*, *MYORG (X1)*, *Desmin*, *Myl3*, *MTMR7*, *MYOC*, *AFAP1*, *PHACTR3*, *ABLIM2*, *SMARCD3*, *Synpo2*, *Olfm3*, *Ablim1*, *SYNPO2L*, *MUSK*, *ACTN2*, *TNNT3*, *SSX2IP*, *Milp*. It suggested that BM-MSCs seemed to be converted into myogenic phenotypic under 5-Aza treatment.

GO and KEGG enrichment was performed to further explore the function of 2402 identified DEGs. Plasma membrane-related terms, binding-related terms, and cell cycle and differentiation-related terms were enriched in CC, MF, and BP respectively. They are all associated with cell differentiation. In the KEGG pathways analysis, the PI3K-Akt signaling pathway and TGF-β signaling pathway were enriched. These two signaling pathways were related to cell differentiation. Furthermore, muscle-related pathways were also enriched, such as arrhythmogenic right ventricular cardiomyopathy, dilated cardiomyopathy, and hypertrophic cardiomyopathy. Thus, we believed that 5-Aza has the potential to induce myogenic differentiation of chicken BM-MSCs, and it seems to induce BM-MSCs into cardiomyocytes. Together, these results provide important insight that 5-Aza probably induces myogenic differentiation of BM-MSCs, which lays a foundation for constructing a myogenic differentiation model in chicken BM-MSCs.

## 5. Conclusions

To sum up, this is the first investigation to evaluate the myogenic determination and differentiation of chicken BM-MSCs under 5-Aza treatment. Our results demonstrate that 5-Aza-treated BM-MSCs can activate the potential of myogenic differentiation more effectively in comparison to DXMS-HC treatment. GO and KEGG analysis revealed that plasma membrane-related terms, binding-related terms and cell cycle, differentiation-related terms, cell differentiation-related pathways, and cardiomyopathy-related pathways were significantly enriched between 5-Aza-treated BM-MSCs and control. Our study provides an experimental basis for establishing a myogenic differentiation model in BM-MSCs.

## Data Availability

Not applicable.

## Supplementary Materials

The following supporting information can be downloaded at: https://www.mdpi.com/article/10.3390/ani12121531/s1, Figure S1: Morphologic observation and growth curve of BM-MSCs; Figure S2: Morphology observation of BM-MSCs in myogenic induced differentiation; Table S1: List of primers used in RT-PCR and qRT-PCR; Table S2: Quality analyses of transcriptome sequencing and mapping; Table S3: List of muscle-related genes upregulated in 5-Aza-induced BM-MSCs.
