# Supplementary material for: Myogenic Determination and Differentiation of Chicken Bone Marrow-Derived Mesenchymal Stem Cells under Different Inductive Agents
Source: Animals (Basel). 2022 Jun 13;12(12):1531. doi: 10.3390/ani12121531 (PMC9219535; doi:10.3390/ani12121531)
Supplement: Supplementary file 1 [file animals-12-01531-s001.zip › Table S3.pdf]

Table S3. List of muscle-related genes upregulated in 5-Aza-induced BM-MSCs.

id	Gene	Control mean fpkm	Treatment mean fpkm	P Value	FDR	Log2(fc)	Description
ncbi_396211	MYH11	7.430	40.073	0.000	0.000	2.431	Myosin heavy chain 11
ncbi_396237	MYO5A	44.440	90.870	0.000	0.000	1.032	unconventional myosin-Va
ncbi_396445	Mylk	88.943	228.377	0.000	0.000	1.360	myosin light chain kinase, smooth muscle isoform 1
ncbi_419963	MYO1D	48.063	131.570	0.000	0.000	1.453	unconventional myosin-Id isoform X4
ncbi_395534	Myh7b	65.343	133.560	0.000	0.000	1.031	myosin heavy chain, cardiac muscle isoform isoform X1
ncbi_415398	MYO1E	26.500	61.190	0.000	0.000	1.207	unconventional myosin-Ie isoform X1
ncbi_427401	MYORG	0.747	3.330	0.000	0.000	2.157	myogenesis-regulating glycosidase
ncbi_419690	MYOM3	0.690	2.700	0.000	0.000	1.968	myomesin-3 isoform X1
ncbi_395805	MYOM1	0.077	0.587	0.000	0.000	2.936	myomesin-1
ncbi_430027	MYO5C	0.237	0.630	0.000	0.000	1.412	unconventional myosin-Vc isoform X1
ncbi_431653	MYORG (X1)	0.013	0.320	0.000	0.000	4.585	myogenesis-regulating glycosidase-like isoform X1
ncbi_395906	DES	0.123	0.577	0.000	0.000	2.225	desmin
ncbi_396067	Myl3	0.487	1.390	0.000	0.001	1.514	myosin light chain 1, cardiac muscle isoform X1
ncbi_422731	MTMR7	0.123	0.303	0.003	0.007	1.298	myotubularin-related protein 7 isoform X1
ncbi_424391	MYOC	0.223	0.513	0.017	0.033	1.201	myocilin
ncbi_374034	AFAP1	86.873	220.540	0.000	0.000	1.344	actin filament-associated protein 1 isoform a
ncbi_770781	PHACTR3	1.033	10.410	0.000	0.000	3.333	phosphatase and actin regulator 3 isoform X1
ncbi_422866	ABLIM2	4.433	18.797	0.000	0.000	2.084	actin-binding LIM protein 2 isoform X1
ncbi_430336	SMARCD3	3.740	9.363	0.000	0.000	1.324	SWI/SNF-related matrix-associated actin-dependent regulator of chromatin subfamily D member 3 isoform X1
ncbi_428768	Synpo2	0.237	2.103	0.000	0.000	3.152	synaptopodin-2
ncbi_424461	Olfm3	2.477	7.203	0.000	0.000	1.540	noelin-3 isoform X1
ncbi_423911	Ablim1	0.147	0.887	0.000	0.000	2.596	actin-binding LIM protein 1 isoform X9
ncbi_423743	SYNPO2L	0.007	0.230	0.000	0.000	5.109	synaptopodin 2-like protein isoform X1
ncbi_373897	MUSK	0.117	0.560	0.000	0.000	2.263	muscle, skeletal receptor tyrosine protein kinase precursor
ncbi_396263	ACTN2	0.047	0.453	0.000	0.000	3.280	alpha-actinin-2

ncbi_395761	TNNT3	0.013	0.193	0.021	0.038	3.858	troponin T, fast skeletal muscle isoforms isoform X4
ncbi_424532	SSX2IP	0.020	0.123	0.027	0.049	2.624	afadin- and alpha-actinin-binding protein isoform X1
ncbi_421889	Mlip	0.001	0.183	0.003	0.006	7.518	muscular LMNA-interacting protein isoform X1
