# Supplementary material for: Myogenic Determination and Differentiation of Chicken Bone Marrow-Derived Mesenchymal Stem Cells under Different Inductive Agents
Source: Animals (Basel). 2022 Jun 13;12(12):1531. doi: 10.3390/ani12121531 (PMC9219535; doi:10.3390/ani12121531)
Supplement: Supplementary file 1 [file animals-12-01531-s001.zip › Figure S1.pdf]

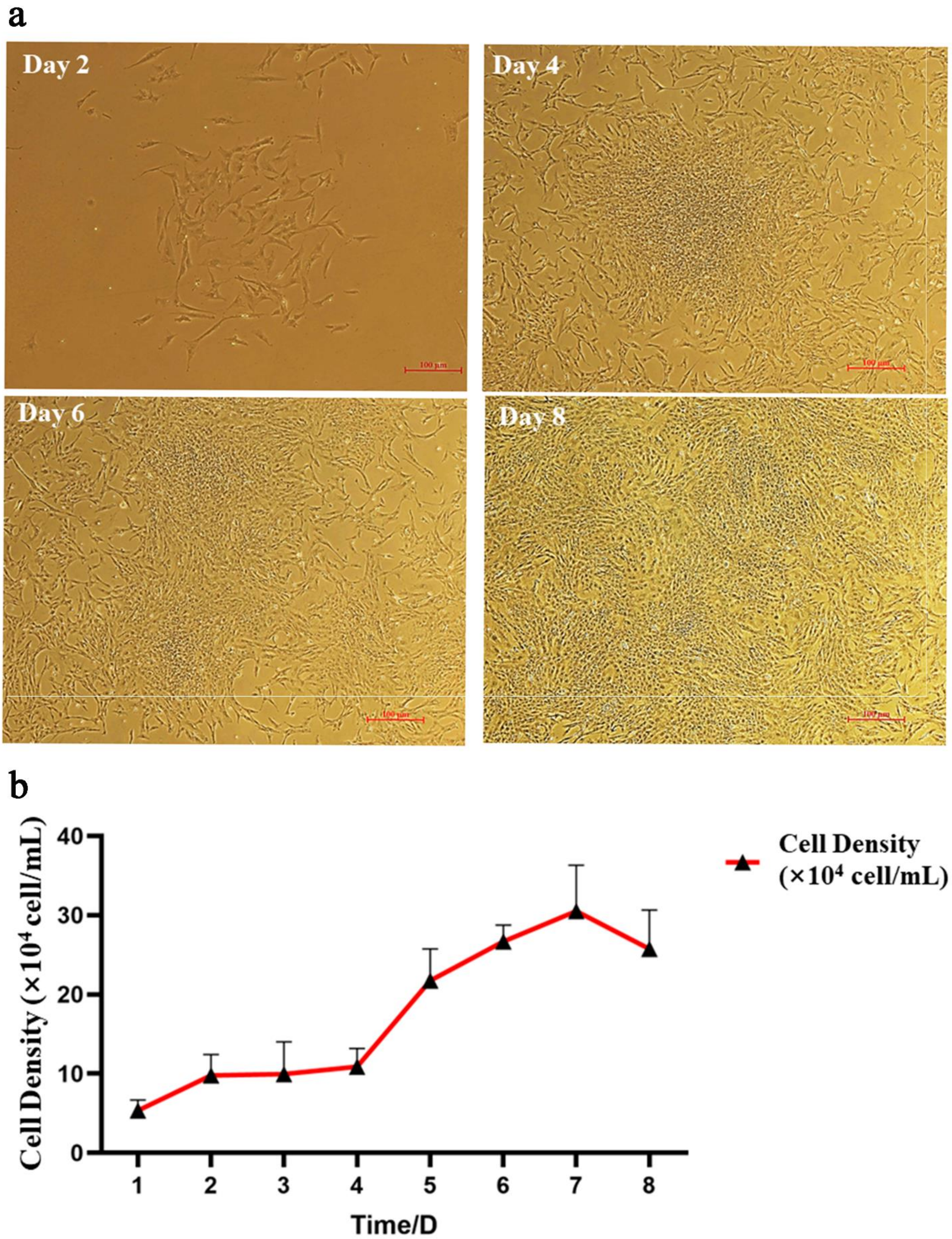

Figure S1. Morphologic observation and growth curve of BM−MSCs in passage 1. (**a**) Morphologic observation of BM−MSCs on different days (2, 4, 6, 8) and cells reached 50% confluence in 4 days, 80% confluence in 6 days, and 100% confluence in 8 days. (**b**) BM−MSCs showed a latent phase of 1–4 days and a logarithmic growth phase for 4–7 days. When culture was up to 8 days, the number of cells began to decrease.
