# Supplementary material for: Myogenic Determination and Differentiation of Chicken Bone Marrow-Derived Mesenchymal Stem Cells under Different Inductive Agents
Source: Animals (Basel). 2022 Jun 13;12(12):1531. doi: 10.3390/ani12121531 (PMC9219535; doi:10.3390/ani12121531)
Supplement: Supplementary file 1 [file animals-12-01531-s001.zip › Figure S2.pdf]

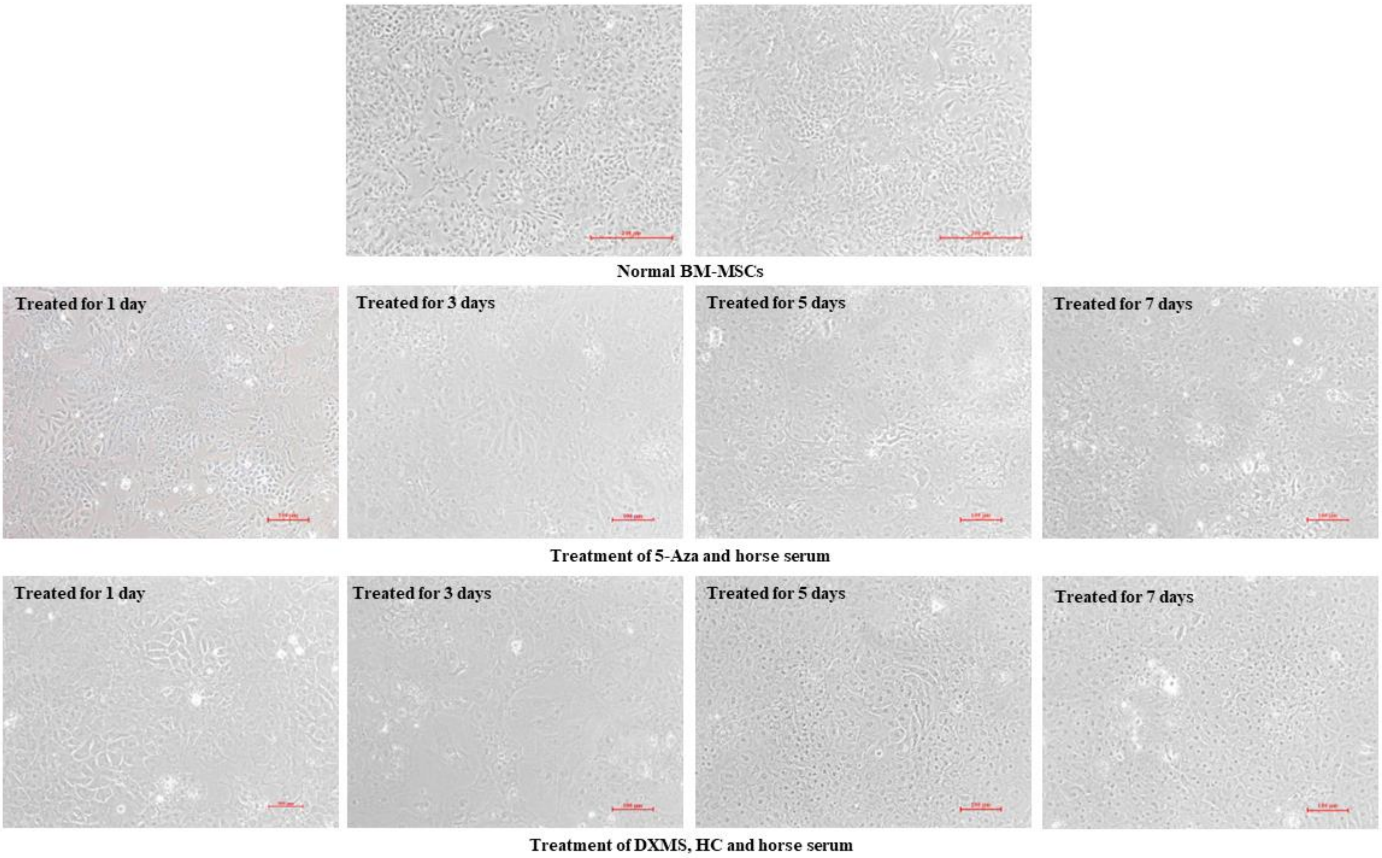

Figure S2. Morphology observation of BM-−MSCs in myogenic induced differentiation. Both treatments resulted in a dramatic morphological change in cell morphology. However, both cultures seemed not to form obvious myotubes.
