# Supplementary material for: Myogenic Determination and Differentiation of Chicken Bone Marrow-Derived Mesenchymal Stem Cells under Different Inductive Agents
Source: Animals (Basel). 2022 Jun 13;12(12):1531. doi: 10.3390/ani12121531 (PMC9219535; doi:10.3390/ani12121531)
Supplement: Supplementary file 1 [file animals-12-01531-s001.zip › Table S1.pdf]

Table S1. List of primers used in RT-PCR and qRT-PCR.

Gene	Primers Sequence（5′---3′）	Product Length (bp)	Usage
CD73	F: TTGCAGGTTGGAGGTATCCAC<br>R: CAAAGAGAAGTGTGTGCGCC	791	RT-PCR
CD71	F: CCCAGGCTTCCCTTCGT<br>R: GGGCTCCAATCACAACATAC	305	RT-PCR
CD90	F: TCAGTGAGAGTGGGGAGAGG<br>R: GGGCTCCAATCACAACATAC	181	RT-PCR
CD29	F: GAACGGACAGATATGCAACGG<br>R: TAGAACCAGCAGTCACCAACG	300	RT-PCR
CD44	F: CATCGTTGCTGCCCTCCT<br>R: ACCGCTACACTCCACTCTTCAT	290	RT-PCR
CD45	F: CACTGGGAATCGAGAGGAAA<br>R: CTGGTCTGGATGGCACTTTT	574	RT-PCR
CD31	F: CAGGCAAAGGAGACGCACGAT<br>R: CTTCTGGCAGCTCACAACGT	210	RT-PCR
CD34	F: GTGCCACAACATCAAAGACG<br>R: GGAGCACATCCGTAGCAGGA	239	RT-PCR
GAPDH	F: CAACTTTGGCATTGTGGAGG<br>R: CGCTGGGATGATGTTCTGG	130	qRT-PCR
Desmin	F: GCTCAATGTCAAGATGGCCC<br>R: CTGGGCTGGTCTCTCGGAAA	134	qRT-PCR
MyHc	F: CTCCTCACGCTTTGGTAA<br>R: TGATAGTCGTATGGGTTGGT	213	qRT-PCR
Myomaker	F: TGGGTGTCCCTGATGGC<br>R: CCCGATGGGTCCTGAGTAG	135	qRT-PCR
MyoD1	F: CGACGGCATGATGGAGTAC<br>R: CAGTCGAGGCTGGAAACAA	90	qRT-PCR
MyoG	F: AACCAGCAGGAGCGTGAGC<br>R: CTGGGTGCAGCAGGTTGTG	68	qRT-PCR
CEBPA	F: GACAAGAACAGCAACGAGTACC<br>R: CCTGAAGATGCCCCGCAGAGT	195	qRT-PCR
CEBPB	F: AACCTGTCCACCTCGTCCT<br>R: CCAAGACTTTGTGCTGCGTC	241	qRT-PCR
PPARG	F: CCAGCGACATCGACCAGTTA<br>R: AGAGCGAAACTGACATCGCT	275	qRT-PCR
β-actin	F: GATATTGCTGCGCTCGTTG<br>R: TTCAGGGTCAGGATACCTCTT	178	qRT-PCR
