# Supplementary material for: Myogenic Determination and Differentiation of Chicken Bone Marrow-Derived Mesenchymal Stem Cells under Different Inductive Agents
Source: Animals (Basel). 2022 Jun 13;12(12):1531. doi: 10.3390/ani12121531 (PMC9219535; doi:10.3390/ani12121531)
Supplement: Supplementary file 1 [file animals-12-01531-s001.zip › Table S2.pdf]

Table S2. Quality analyses of transcriptome sequencing and mapping.

Sample	Raw reads	Clean reads	Error rate (%)	Clean base (bp)	Q20 (%)	Q30 (%)	GC content (%)	Total mapped
Control-1	48075044	47845636 (99.52%)	0.05 %	7149807321	97.56 %	93.19 %	47.00 %	44752589 (93.73%)
Control-2	46131780	45908026 (99.51%)	0.05 %	6858957950	97.65 %	93.44 %	47.11 %	42962100 (93.80%)
Control-3	63671684	63355808 (99.50%)	0.05 %	9465494334	97.53 %	93.17 %	47.09 %	59226313 (93.74%)
Treatment-1	49584556	49358426 (99.54%)	0.04 %	7377333198	97.65 %	93.44 %	46.68 %	46050399 (93.51%)
Treatment-2	42558286	42357262 (99.53%)	0.05 %	6332782877	97.65 %	93.42 %	46.59 %	39481176 (93.42%)
Treatment-3	51759574	51518282 (99.53%)	0.05 %	7698048137	97.76 %	93.67 %	46.54 %	48069930 (93.53%)

Note: Control represents BM-MSCs treated with nothing. Treatment represents BM-MSCs under 5-Aza treatment for 5 days. Raw reads mean the number of sequencing sequences generated from library construction while clean reads mean the number of sequences filtered by sequencing data. Q20 and Q30 represent the percent of sequenced bases that have a predicted quality score of 20 and 30 after mapping. GC content means the dependence between fragment counts (read coverage). Total mapped describes the statistics of the sequencing sequences that can be located on the reference genome.
